# Future uncertainties in the development of clinical cerebral oximetry

**DOI:** 10.3389/fphys.2013.00360

**Published:** 2013-12-18

**Authors:** Hilary P. Grocott, Sophie N. Davie

**Affiliations:** ^1^Department of Anesthesia & Perioperative Medicine, University of ManitobaWinnipeg, MB, Canada; ^2^Department of Surgery, University of ManitobaWinnipeg, MB, Canada

**Keywords:** cerebral oximetry, cardiac surgery, near infrared spectroscopy, postoperative outcome, brain protection

Since the first report in 1954 outlining the *in vivo* use of a double-beam spectrophotometer to measure the absorption of near-infrared light by various light absorbing pigments (or chromophores) involved in the mitochondrial respiratory chain (Chance, 1954), substantive advancements have been made in the use of near-infrared spectroscopy (NIRS) to monitor the oxidative state of various human tissues. Indeed, when Chance first reported the measurement of cytochrome c oxidase in yeast cells (Chance, 1954), he likely had little inclination that this would one day lead to the development of the modern day cerebral oximeter, an increasingly utilized monitor aimed at optimizing patient outcomes in a range of surgical settings. However, it was almost 25 years later that Jöbsis published a series of investigations in animals (and in human volunteers) demonstrating that blood flow related changes in brain oxygenation could be monitored non-invasively. These studies established that NIRS could be used to monitor regional brain oxygen saturation (rSO_2_) in a potentially clinically useful manner (Jobsis, 1977). However, as is the case with many clinical developments, it took decades of further advancements before the first cerebral oximeter was approved by the United States Food and Drug Administration (Widman, 1997). Indeed, now more than 20 years since its first approval (and 60 years after the seminal work by Chance), we are arguably at a cross roads in the further development and understanding of how to use cerebral oximetry in clinical practice. Although substantial potential exists for NIRS-associated advancements in patient monitoring (and related improvements in outcome) in a wide variety of clinical situations, there are a number of uncertainties that could prevent these devices from reaching their full potential.

Although there are hundreds of reports outlining numerous plausible uses for cerebral oximetry, notably in the operating room and intensive care unit (Grocott et al., 2010; Ghosh et al., 2012; Zheng et al., 2013), we are still years from having large scale, randomized controlled clinical trials definitively examining the full potential clinical benefit of this device. The results of smaller trials, if they are to be confidently believed, need to be replicated and corroborated on a much larger scale. Indeed, there are a number of reasons for the considerable uncertainty regarding the capability of cerebral oximeters, despite substantive promise. Inter-device variability, variability in oxygen saturation targets and thresholds, issues related to absolute vs. relative saturation changes, along with studies limited by their observational nature, have all contributed to this current lack of confidence. Understanding the true clinical value of this device is further obscured by wide inter-study variability.

With respect to the variability in technology amongst the individual devices, it is not entirely certain whether information gained from one manufacturer's device can be appropriately compared to that of another. The differences in technology, including the choice of light source (i.e., laser vs. light emitting diodes) and specific wavelengths, the various proprietary absorption and processing algorithms, as well as the validation sequences used, all contribute to questions of their broader comparability (Ghosh et al., 2012). Variability might also exist with respect to how the devices account for differences in skin pigmentation (Bickler et al., 2013). Furthermore, the manner in which these devices handle issues related to spatial resolution could have the largest impact on whether rSO_2_ measurements are affected by extracranial contamination (Davie and Grocott, 2012). That is, how much non-cerebral saturation signal from the superficial tissues, contaminating the deeper cerebral saturation signal, does each of these devices contain? Indeed, we recently determined, using an experimental design that allowed the separation of scalp oxygen saturation from that of cerebral oxygen saturation, that the scalp contamination can lead to as much as a 17% change in the NIRS signal. Importantly, newer generation devices were much less prone to the contamination. Comparative studies using more than one technology will be needed to determine if the differences in technology are clinically relevant.

Optimizing outcome is predicated on understanding what brain oxygen saturation thresholds are worrisome enough to clinically warrant intervention. Reports thus far have substantive inconsistencies as to what the optimal cerebral saturation target should be—which leads us to the question: what is the desaturation threshold, that when crossed, increases the risk of adverse outcomes? (Murkin et al., 2007; Hemmerling et al., 2008; Kazan et al., 2009; Fischer et al., 2011; Heringlake et al., 2011; Tang et al., 2012). Specifically, is there an *absolute* saturation threshold at which there is an increased risk of adverse clinical outcomes, such has been demonstrated with jugular bulb desaturation in carotid endarterectomy surgery (Moritz et al., 2008), or is there a *relative* change (from a pre-determined baseline saturation) that is more important to signal impending danger? Furthermore, if it is a relative change, how much of a relative change? Defining desaturation is further complicated by the influence of oxygen supplementation on relative changes in cerebral oxygenation. For example, breathing 100% oxygen can elevate baseline rSO_2_ and artificially widen the difference between baseline rSO_2_ and the lowest rSO_2_ value during a surgical procedure (Bussieres et al., 2012). Finally, it is uncertain as to whether there is a “dose response” with respect to cerebral desaturation. That is, does a brief but substantial desaturation portend a worse outcome compared to a lesser degree of desaturation occurring for a more prolonged period of time? Early data examining the relationship between the area under the curve for various rSO_2_ thresholds suggests the latter may be the case (Fischer et al., 2011). Whether it is an absolute or relative change in rSO_2_ that is important will only be determined by analyzing both variables in adequately sized observational trials (see below).

The interventional algorithms used to optimize saturation are also incompletely described with respect to which of the commonly used interventions (FiO_2_, PaCO_2_, blood pressure, cardiac output, hemoglobin) are considered best to optimize rSO_2_ (Denault et al., 2007). Each one of these interventions could conceivably improve outcome, or make it worse. Increasing the PaCO_2_, blood pressure and cardiac output could increase cerebral blood flow while augmenting the FiO_2_ and hemoglobin (with transfusion) could also increase the oxygen content. Combined, this would increase overall cerebral oxygen delivery. Conversely, there is the potential for substantive morbidity related to inappropriate transfusion, targeted hypercapnia and/or hypertension. For example, erroneously increasing the PaCO_2_ above the normal physiologic level in the setting of cardiopulmonary bypass (CPB) could lead to increases of cerebral blood flow in excess of what is needed for cerebral metabolism, with a subsequent increased delivery of CPB-related air bubbles and particulate debris. This is similar to the use of pH-stat blood gas management, where the addition of CO_2_ to the CPB fresh gas flow (thus correcting the CO_2_ for hypothermic temperature) has been associated with worse postoperative cognitive outcomes (Patel et al., 1996). Ultimately, the safety of various interventional algorithms will be borne out from prospective randomized controlled trials of large size and multi-center nature.

Arguably, one of the greatest limitations thus far in our understanding of cerebral oximetry has been the failure to confidently delineate which clinical outcomes have relevance to rSO_2_ measurements. That is, one would intuitively expect that because it is a direct cerebral oximeter, neurologic outcomes themselves would have the best relationship to brain oxygen saturation. However, a cogent argument can easily be made regarding the biologic plausibility that non-cerebral outcomes could be better related to measurements of cerebral saturation. Murkin has logically argued that the brain serves as an “index organ” for other tissue saturation (Murkin, 2011). However, the brain's inherent protective mechanisms (such as metabolic and pressure related autoregulation) suggest that if brain desaturation does occur, it is likely that other tissues have long since desaturated (Boston et al., 2001; Grocott, 2011). That is, the brain in effect is the last organ to be compromised, and in some respects is not an early warning system such as a canary in the coalmine (Grocott, 2012), but just the opposite (i.e., a late indicator of trouble).

Determining which endpoints to target in outcome studies employing cerebral oximetry-guided intervention could be the largest roadblock to the clinical progress of cerebral oximetry. Our understanding of how oximetry could impact patient outcomes has been limited by the variable endpoints outlined in multiple, but mostly small, observational studies. These outcomes have been so diverse that there is considerable uncertainty as to which ones could confidently be the focus in larger trials. We would argue that there is still a desperate need for a large prospective observational trial involving at least 1000 patients, if not more (an arbitrary number, but seemingly large enough study), to determine which outcomes may be related to desaturation. Only then would it be possible to design a randomized controlled trial to investigate the ability of an interventional algorithm, aimed at restoring cerebral saturation, to modify the previously determined target outcomes. This could also serve to delineate a potential saturation threshold to maintain (such as a relative or absolute change). A good example of this is the recent work that we performed in a small observational study (*n* = 109) in cardiac surgery (Arenson et al., 2013). Firstly, we demonstrated that a somewhat conservative threshold (of an absolute saturation of less than 50%) showed a far stronger relationship to adverse outcomes [such as acute kidney injury (AKI)] than any of the relative changes from baseline. Furthermore, and as previously hypothesized, it was the non-neurologic outcomes that had substantive relationship to desaturation, with renal dysfunction demonstrating the strongest relationship. This is consistent with other recent work demonstrating that when patients were managed at low blood pressures (i.e., those below cerebral autoregulatory thresholds as defined by cerebral oximetry), they had an increased incidence of AKI (Ono et al., 2013).

Thus, the field of cerebral oximetry research is still relatively young and contains as many questions as it does certainty. In order to move forward in an expedited and confident fashion, taking advantage of the last 60 years of research in cerebral NIRS, we will need to reconsider our approach regarding further clinical development. Otherwise, we risk wasting this potentially valuable technology, and most importantly, depriving our patients the benefit of improved clinical outcomes with its universal use.
